# RFRP3 influences basal lamina degradation, cellular death, and progesterone secretion in cultured preantral ovarian follicles from the domestic cat

**DOI:** 10.7717/peerj.7540

**Published:** 2019-08-23

**Authors:** Kathryn Wilsterman, George E. Bentley, Pierre Comizzoli

**Affiliations:** 1 Integrative Biology, University of California, Berkeley, Berkeley, CA, United States of America; 2 Helen Wills Neuroscience Institute, University of California, Berkeley, Berkeley, CA, United States of America; 3 Smithsonian Conservation Biology Institute, Washington, DC, United States of America

**Keywords:** NPVF, Ovary, GnIH, Steroid, HPG, Reproduction

## Abstract

The hypothalamic neuropeptide RFRP3 can suppress hypothalamic GnRH neuron activation and inhibit gonadotropin release from the anterior pituitary. RFRP3 is also produced locally in the ovary and can inhibit steroidogenesis and follicle development in many vertebrates. However, almost nothing is known about the presence and regulatory action of RFRP3 in gonads of any carnivore species. Such knowledge is important for developing captive breeding programs for endangered carnivores and for inhibiting reproduction in feral species. Using the domestic cat as a model, our objectives were to (1) demonstrate the expression of feline RFRP3 (fRFRP3) and its receptor in the cat ovary and (2) assess the influence of fRFRP3 on ovarian follicle integrity, survival, and steroidogenesis *in vitro*. We first confirmed that fRFRP3 and its receptors (NPFFR1 and NPFFR2) were expressed in cat ovaries by sequencing PCR products from ovarian RNA. We then isolated and cultured preantral ovarian follicles in the presence of 10 or 1 µM fRFRP3 + FSH (1 µg/mL). We recorded the percentage of morphologically viable follicles (basal lamina integrity) over 8 days and calculated percentage survival of follicles on Day 8 (using fluorescent markers for cell survival and death). Last, we quantified progesterone accumulation in media. 10 µM fRFRP3 had no observable effect on viability, survival, or steroid production compared to follicles exposed to only FSH. However, 1 µM fRFRP3 decreased the percentage of morphologically viable follicles and the percentage of surviving follicles on Day 8. At the same time, 1 µM fRFRP3 increased the accumulation of progesterone in media. Our study shows, for the first time, direct action of RFRP3 on the follicle as a functional unit, and it is the first in a carnivore species. More broadly, our results support a conserved, inhibitory action of RFRP3 on ovarian follicle development and underscore the importance of comparative functional studies.

## Introduction

In spite of their name, neuropeptides are synthesized in and act on many peripheral tissues in addition to their classical actions in the nervous system (McGuire & Bentley, 2010). In part due to persistent biases in how these peptides are studied, we still have a rudimentary understanding of how these neuropeptides evolved to play disparate central and peripheral roles. In many cases we do not even have a comprehensive understanding of their function or importance outside of the brain (Bentley et al., 2017).

While some neuropeptides have peripheral effects that serve distinct functions from their role in the central nervous system (e.g., the neuropeptide gonadotropin-releasing hormone (GnRH); Iwakoshi-Ukena et al., 2004; Bentley et al., 2017), other neuropeptides exhibit putatively concordant function in the central nervous system and periphery. One such example is the nonapeptide RF-amide related peptide 3 (RFRP3). Originally discovered in birds (Tsutsui et al., 2000), orthologs of RFRP3 (variously known as GnIH and LPXRFa) perform similar functions in other vertebrates. For clarity, we use RFRP3 to refer to this family of genes throughout the manuscript. RFRP3 inhibits activity of the vertebrate reproductive axis via direct action in the hypothalamus, on the anterior pituitary, and in the gonads (Bentley et al., 2017). In the hypothalamus, RFRP3 can inhibit the activity of GnRH cells (Murakami et al., 2008; Clarke et al., 2008; Anderson et al., 2009; Kadokawa et al., 2009; Ducret, Anderson & Herbison, 2009; Bentley, Tsutsui & Kriegsfeld, 2010; Son et al., 2016). Hypothalamic RFRP3 can also directly influence pituitary release of gonadotropins (Osugi et al., 2004; Tsutsui, Bentley & Ciccone, 2015; Kriegsfeld et al., 2006; Tsutsui et al., 2006; Anderson et al., 2009; Bentley, Tsutsui & Kriegsfeld, 2010). Furthermore, RFRP3 is found in ovarian tissues across nearly all vertebrate taxa studied to-date; of 18 species examined from lampreys to humans, only the grass puffer lacks ovarian RFRP3 (Table 1). However, there are far fewer studies that have assessed function of RFRP3 in the ovary; of the 17 species in which ovarian RFRP3 has been identified, only seven have had any function evaluated (Table 1).

**Table 1:** Observational and functional studies of RFRP3 and orthologs in vertebrate ovaries.

**Cit.**	**Class**	**Order**	**Species/Strain**	**Approach**	**Summary of findings**
**Observational findings**					
Osugi et al. (2012)	Hyperoartia	Petromyzontiformes	*Petromyzon marinus*	PCR	RFRP3 expressed in the ovary
Biran et al. (2014)	Osteichthyes	Cichliformes	*Oreochromis niloticus*	PCR	RFRP3 and receptor expressed in the ovary
Qi et al. (2013)	Osteichthyes	Cypriniformes	*Carassius auratus*	*in situ*	RFRP3 receptor expression abundant in early-stage follicles
Zhang et al. (2010)	Osteichthyes	Cypriniformes	*Danio rerio*	PCR	RFRP3 and RFRP3-R expressed in the ovary
Corchuelo et al. (2017)	Osteichthyes	Cypriniformes	*Danio rerio*	PCR/*in situ*	RFRP3 expression in the ovary highest during primary growth of follicles, and lower during later stages of follicle growth (PCR); RFRP3 expressed in the granulosa cells of vitellogenic follicles (*in situ*)
Paullada-Salmerón et al. (2016)	Osteichthyes	Perciformes	*Dicentrarchus labrax*	PCR	RFRP3 expressed in the ovary (very low)
Wang et al. (2018)	Osteichthyes	Pleuronectiformes	*Cynoglossus semilaevis*	PCR	RFRP3 expressed in ovary; RFRP3 expression ten-times more intense during previtellogenesis relative to other stages of ovary maturation
Shahjahan et al. (2011)	Osteichthyes	Tetraodontiformes	*Takifugu niphobles*	PCR	RFRP3 expression **absent** in the ovary
Singh et al. (2008)	Reptilia	Squamata	*Calotes versicolor*	IHC/slot blot	RFRP3-ir highest in the stroma of resting-phase ovaries (IHC); RFRP3-ir present in GCs of the dominant follicle during recrudescence and the oocyte of the dominant follicle during folliculogenesis (IHC); RFRP3 protein abundance increases across vitellogenesis, ovulation, and regression, with abundance highest in resting-phase ovaries (slot blot)
Bentley et al. (2008)	Aves	Galliformes	*Coturnix japonica*	PCR	RFRP3 and RFRP3-R expressed in the ovary (PCR);
Maddineni et al. (2008)	Aves	Galliformes	*Gallus gallus domesticus*/ White Leghorn	PCR	RFRP3-R expression in TCs decreases with follicle maturation; RFRP3-R is more abundant in GCs of all follicles than in TCs
Bentley et al. (2008)	Aves	Passeriformes	*Zonotrichia leucophrys Gambelii*	*in vivo* & *in vitro* receptor fluorography/IHC	Bindings sites for RFRP3 found in the GCs of ovarian follicles (*in vivo* & *in vitro* receptor fluorography); RFRP3-ir localized to ovarian GCs (IHC)
Bentley et al. (2008)	Aves	Passeriformes	*Sturnus vulgaris*	*in vivo* receptor fluorography/PCR/IHC	Bindings sites for RFRP3 in the GCs of ovarian follicles (*in vivo* receptor fluorography); RFRP3 and RFRP3-R expressed in the ovary (PCR); RFRP3-ir localized to ovarian GCs (IHC)
Li et al. (2014)	Mammalia	Artiodactyla	*Ovis aries*/Dorper ×Hu F1	PCR/IHC	RFRP3 expression in the ovary (PCR); RFRP3 expressed in oocytes (IHC); RFRP3 expressed in GCs tertiary follicles only (IHC)
Li et al. (2012)	Mammalia	Artiodactyla	*Sus scrofa domesticus/* Suzhong (PCR) Large white cross-bred (IHC)	PCR/IHC	RFRP3 expression in ovary most abundant during proestrous and least abundant during estrous (PCR); RFRP3-R expression in the ovary most abundant at estrous and least abundant during diestrous (PCR); Most intense RFRP3-ir found in the GCs during estrous; RFRP3-ir also found in TCs, and CL (IHC); RFRP3-R-ir most intense in the TCs and GCs of mature follicles during estrous; RFRP3-R-ir also found in the CL (IHC)
Fang et al. (2014)	Mammalia	Artiodactyla	*Sus scrofa domesticus*/ Yorkshire	PCR	RFRP3 expressed in the pubertal ovary
Singh et al. (2011)	Mammalia	Rodentia	*Mus musculus domesticus*/ Parkes	IHC	RFRP3-ir greatest in non-luteolytic CLs and the GCs/TCs of mature follicles
	Mammalia Rodentia	*Rattus norvegicus domesticus/*	IHC	RFRP3-ir present in the interstitial tissues and GCs of antral follicles; low RFRP3-ir in the CL
Squicciarini et al. (2018)	Mammalia	Rodentia	*Rattus norvegicus domesticus/* Sprague-Dawley	IHC	RFRP3-ir present in the interstitial tissues and GCs of antral follicles; low RFRP3-ir in the CL
Oishi et al. (2012)	Mammalia	Primates	*Homo erectus*	IHC	RFRP3 and RFRP3-R present in CLs and GCs/TCs of pre-ovulatory follicles
McGuire & Bentley (2010)	Mammalia	Primates	*Macaca mulatta*	*in situ*	RFRP3 and RFRP3-R expressed in GCs and oocytes
**Functional findings**					
Wang et al. (2017)	Osteichthyes	Perciformes	*Epinephelus coioides*	Ovarian explant	RFRP3(I) increases expression of StAR and 3βHSD; RFRP3(II) increases expression of LHR
Singh et al. (2008)	Reptilia	Squamata	*Calotes versicolor*	Ovarian explant	RFRP3 decreases expression of GnRH-R in the ovary
Maddineni et al. (2008)	Aves	Galliformes	*Gallus gallus domesticus/* White Leghorn	Isolated granulosa cells	RFRP3 decreases cell survival at 10 and 1000 nM, but only in the absence of FSH
Li et al. (2013)	Mammalia	Artiodactyla	*Sus scrofa domesticus/* Large White	Isolated granulosa cells	RFRP3 at 1000 and 0.1 nM groups exerts non-dose dependent inhibition of E_2_ production; RFRP3 has no effect on P_4_ production; RFRP3 exerts dose-dependent inhibition of ERK and PCNA expression.
Wang, Li & Hu (2018)	Mammalia	Artiodactyla	*Sus scrofa domesticus/* Unknown	Isolated granulosa cells	RFRP3 dose-dependently inhibits GC proliferation; RFRP3 induces cell cycle arrest in the G2/M phase
Singh et al. (2011)	Mammalia	Rodentia	*Mus musculus domesticus*/ Parkes	Ovarian explant	RFRP3 dose-dependently inhibits GnRH-1-R expression in the ovary
Singh, Krishna & Tsutsui (2011)	Mammalia	Rodentia	*Mus musculus domesticus*/ Parkes	Ovarian explant	RFRP3 inhibits P_4_ production; RFRP3 decreases the expression of StAR and 3βHSD
Squicciarini et al. (2018)	Mammalia	Rodentia	*Rattus norvegicus domesticus/* Sprague-Dawley	Ovarian explant	RFRP3 decreases P_4_ & T production only under the presence of LH
Squicciarini et al. (2018)	Mammalia	Rodentia	*Rattus norvegicus domesticus/* Sprague-Dawley	Peristaltic pump	RFRP3 treatment is associated with larger CLs in the ovary
Oishi et al. (2012)	Mammalia	Primates	*Homo erectus*	Isolated granulosa cells	RFRP3 dose-dependently inhibits P_4_ accumulation in the presence of gonadotropins; RFRP3 inhibits StAR expression in the presence of gonadotropins; RFRP3 has no effect on steroid accumulation or steroidogenic gene expression when gonadotropins are not present

**Notes.**

Abbreviations used in table 3βHSD 3-beta-hydroxysteroid dehydrogenase CL corpora lutea *E*_2_ estradiol ERK extracellular regulated kinases FSH follicle stimulating hormone GC granulosa cell RFRP3 RF-amide related peptide3 used throughout table for simplicity for all genes in the LPXRFa, GnIHand RFRP-3 gene family RFRP3-ir RFRP3 immunoreactivity RFRP3-R: RFRP3 receptor RFRP3-R-ir RFRP3 receptor immunoreactivity GnRH-1-R receptor for gonadotropin-inhibitory hormone-1 IHC Immunohistochemistry LH luteinizing hormone LHR luteinizing hormone receptor *P*_4_ Progesterones PCNA proliferating cell nuclear antigen PCR polymerase chain reaction StAR Steroidogenic acute regulatory protein T testosterone TC theca cell

We and others have suggested that the widespread presence of RFRP3 and its receptor in the ovary is indicative of a conserved regulatory action (Tsutsui et al., 2012; Bentley et al., 2017; Kriegsfeld et al., 2018). To date, RFRP3 is known to decrease cell viability and steroidogenic gene expression or steroidogenesis in the ovary of the chicken (Maddineni et al., 2008) and four mammal species (domestic pigs, Li et al., 2013; Parkes mice, Singh, Krishna & Tsutsui, 2011; Sprague-Dawley rats, Squicciarini et al., 2018; and humans, Oishi et al., 2012). Other work in avian systems also indirectly suggests that RFRP3 expression in the ovary is associated with inhibition of the reproductive axis (McGuire, Koh & Bentley, 2013; Ernst, Lynn & Bentley, 2016). However, in the one fish species in which function has been examined, ovarian RFRP3 promoted the transcription of steroidogenic genes (LHR, StAR, 3β-HSD; Wang et al., 2017). Because only seven studies have interrogated any function of the RFRP3 system in the ovaries (Table 1), the discordant actions of RFRP3 among taxa could reflect species-specific variation or broader taxonomic patterns. Further comparative experiments that assess function are needed to evaluate whether ovarian RFRP3 exerts conserved function across species.

The potentially-conserved inhibitory function of RFRP3 in the ovary is exciting because it provides many potential applications spanning reproductive medicine (Squicciarini et al., 2018) to invasive species management (Rhodes, 2017; McCormick & Romero, 2017) and endangered species survival programs (Comizzoli & Holt, 2019). By antagonizing the receptor, we may be able to improve follicle quality or maturation *in vitro*, and genetic tools have the potential to use local overexpression of inhibitory peptides like RFRP3 to provide long-term fertility suppression in feral animals and/or invasive species. Both such applications are particularly pressing for felines: feral cats are an on-going problem for native species (Medina et al., 2011), and the preservation of endangered species requires continued progress in assisted reproductive technologies (Comizzoli & Holt, 2019).

To date, RFRP3 has not yet been examined in any felid or carnivore species. Although many carnivore species and endangered species are difficult to use for large-scale studies of reproductive function, there are tractable, established techniques for studying ovarian function in the domestic cat (*Felis catus*) as a model. In particular, ovarian follicle isolation and *in vitro* culture are a major focus of reproductive physiology research in the domestic cat (Herrick, 2019; Songsasen et al., 2012; Comizzoli & Holt, 2019), and thus this system is well-optimized to serve as a model for studies of effects on follicular function. Although others have suggested that effects of RFRP3 on granulosa cell function *in vitro* will ultimately alter viability of the ovarian follicle and/or oocyte maturation (Maddineni et al., 2008; Wang, Li & Hu, 2018), no one has directly examined the effect of RFRP3 on follicle viability. These advantages identify the domestic cat as an important model for both basic and applied research into RFRP3 function.

We pursued two aims related to understanding function of feline RFRP3 (fRFRP3), and we designed our experiments based on *a priori* predictions. First, we asked whether a functional fRFRP3 signaling system exists in the ovaries of domestic cats. We predicted that fRFRP3 and its receptors, NPFFR1 and NPFFR2, would be expressed in the ovary. Second, we asked whether fRFRP3 impacts ovarian follicle integrity, survival, and steroid production by using an alginate-embedding system to culture isolated follicles (Songsasen et al., 2012; Thongkittidilok et al., 2018; Chansaenroj, Songsasen & Chatdarong, 2019). Based on studies in other mammals, we expected fRFRP3 to dose-dependently inhibit follicle growtth and to result in decreased viability of follicles (Maddineni et al., 2008; Wang, Li & Hu, 2018). We also predicted that fRFRP3 would dose-dependently inhibit progesterone production by follicles (Singh, Krishna & Tsutsui, 2011; Oishi et al., 2012; Li et al., 2013; Squicciarini et al., 2018). To generate an integrated measure of progesterone accumulation, we quantified progesterone using pooled media samples over the culture period from each follicle. We evaluated effects of fRFRP3 in the presence of gonadotropins because effects of RFRP3 have been dependent on gonadotropin presence in primates and rats (Oishi et al., 2012; Squicciarini et al., 2018).

## Materials & Methods

All tissues used in this study were collected with veterinarian permission during surgeries performed for trap, neuter/spay and release programs used to manage local feral cat colonies. The study did not require the approval of the Animal Care and Use Committees of the Smithsonian Institution or University of California, Berkeley.

### Expression of fRFRP3 and receptors in ovaries of the domestic cat

Whole ovarian tissue was flash-frozen in isopentane on dry ice immediately following surgery from routine spays on domestic cats. Tissues were stored on dry ice for transport and then at −80 °C until extraction.

Tissues were sliced at 20 µm using a cryostat, collected into an RNAase-free Eppendorf tube, and stored at −80 °C. Sections collected across the entire ovary (*N* = 2) were represented in each tube. Samples were homogenized and extracted using the Bioline Isolate II RNA Mini Kit (Cat. No. BIO-52073) as per manufacturer instructions. RNA was quality-tested at the University of California, Berkeley Functional Genomics Laboratory using a bioanalyzer. All samples had RIN scores of 8 or higher.

After extraction, 750 ng of RNA from each extraction was reverse-transcribed using the iScript gDNA Clear cDNA Synthesis Kit (Cat. No. 1725035) according to manufacturer instructions. No-RT samples were reverse-transcribed at the same time using 750 ng of RNA and the kit’s No-RT control supermix.

**Figure 1:**
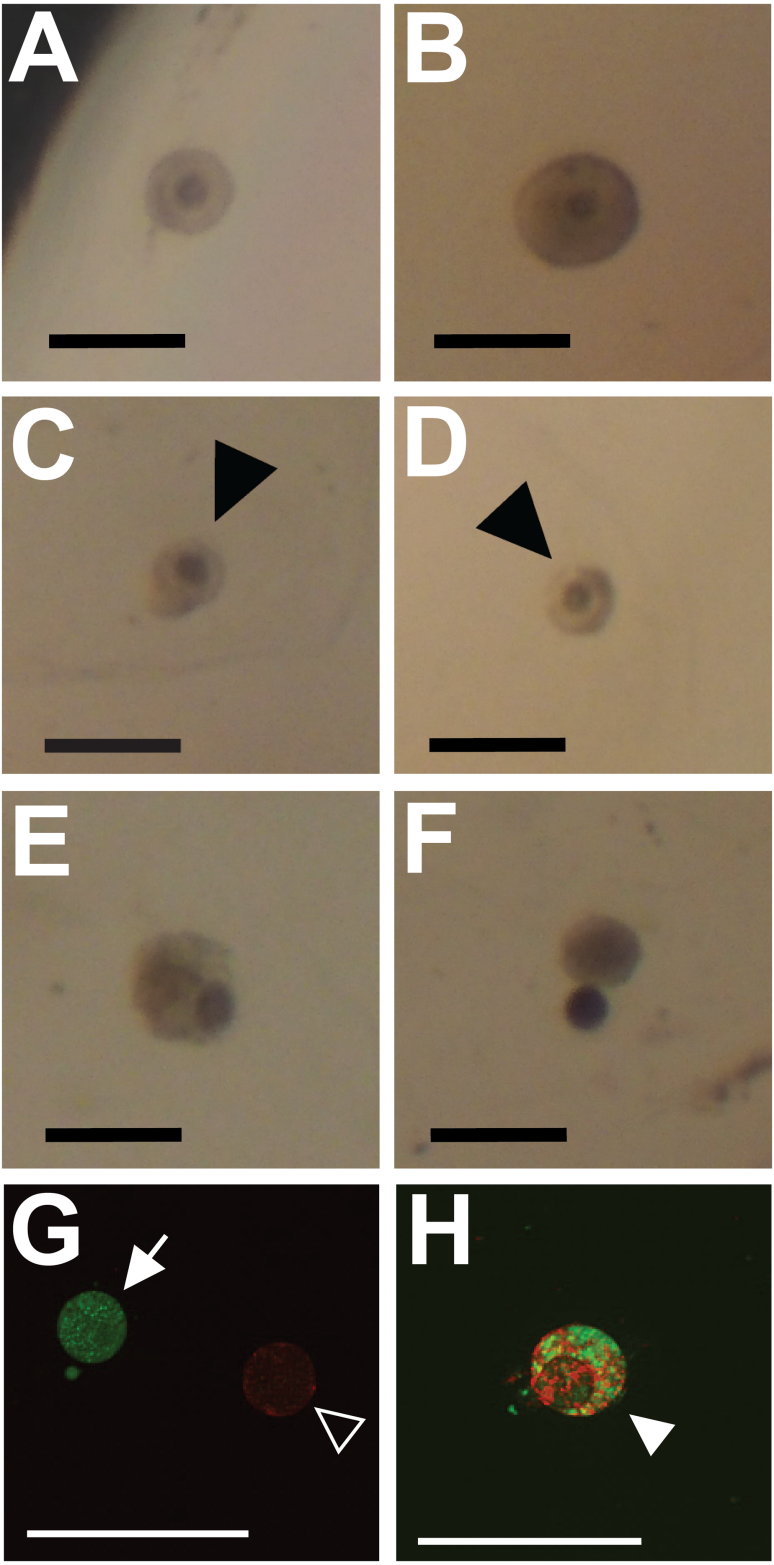
Representative images showing classification of follicles for integrity (A–F) and viability (G, H; cell death) outcome measures. Preantral follicles with intact and relatively even basal lamina were classified as intact (maintaining integrity) (A, B). Follicles with gaps in the basal lamina (arrowheads in C, D), follicles with large sections of the basal lamina missing and the oocyte falling out of the follicle (E, F), or having an absent basal lamina were classified as degraded. (G, H) Fluorescent dyes indicate cell viability in follicles (red for dead, green for alive). Follicles exhibiting only green fluorescence were categorized as alive (filled arrow in G). Follicles with any red fluorescence were categorized as dead or dying (empty arrowhead in H indicates mixed staining, whereas the filled arrowhead in G indicates a follicle fluorescing only in red). Scale bars are equal to 250 µm.

**Figure 2:**
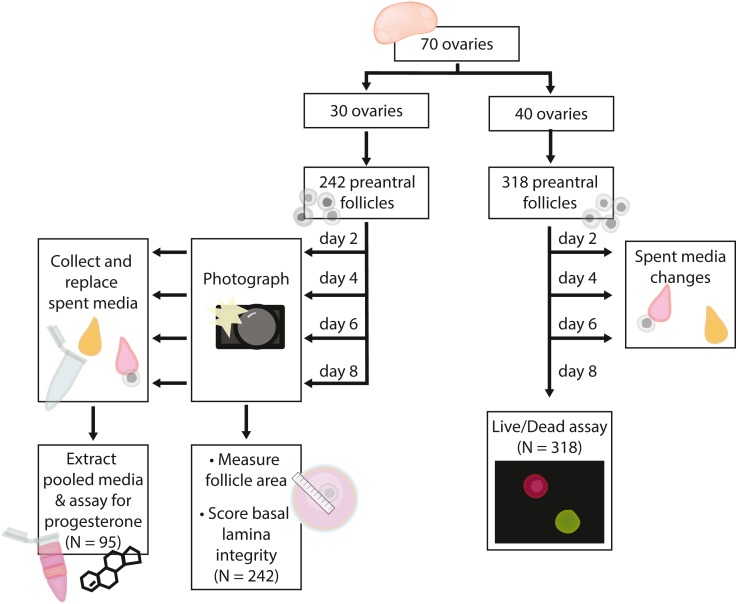
Flow chart summarizing procedures and sample sizes for experiments.

Gene targets were amplified using 18.75 ng of cDNA in a 30 µL reaction containing 0.4 µM forward and reverse primers (Table 2). Other reaction components (TaqPolymerase, reaction buffer, Mg SO_4_, and dNTPs) were added as per manufacturer instructions (Platinum *Taq* DNA Polymerase, High Fidelity; Cat. No. 11304011, Invitrogen). PCR was run for 45 cycles with a 60 °C annealing temperature and 3 min extension. No-RT and no-transcript controls were run alongside experimental samples. No-RT samples omitted the reverse transcriptase during reverse transcription, and no-transcript controls contained water instead of reverse transcription product. The reaction product was run on a 2% agarose gel and imaged. Amplification of the target gene was confirmed using length of the expected product (See Table 2 and Figs. 1 and 2) and sequencing. PCR products were cleaned and sequenced at the University of California, Berkeley DNA Sequencing facility. NPFFR1 sequencing attempts failed to return any sequence, and thus we relied on product length for specificity.

**Table 2:** Primer sequences for PCR amplification.

Target Gene	NCBI Accession Number	Forward	Reverse	Amplicon length
RFRP3	XM_023242159.1	TGATGTCCGGTTTTCACAG	TTTGGACCCCAGTCTTG	118
NPFFR1	XM_023240516.1	CTGTATGCCCACCACTCTCG	CGGAACCTTTCCACAGCAATG	144
NPFFR2	XM_003985291.5	CGGGAAGACTGGCCAAATCA	GTGGGGCACTGTCATCTTGA	141
	XM_023253059.1			

### Tissue handling for cell culture

Whole uterine and ovarian tissue were collected into chilled transport media (Table 3) immediately following routine spays. Within 8 h, ovaries were separated from uterine horns and halved longitudinally. Only follicular ovaries were used for these experiments. Ovaries from pregnant or lactating cats were excluded by assessing uterine status, and ovaries from luteal-phase animals were excluded by presence of corpora lutea in the bisected ovary.

Ovary halves were placed in collection medium (Table 3) and minced using a scalpel blade to release follicles. Preantral follicles were collected from the debris using a 10 µL pipette and transferred to clean, warmed collection medium. Preantral follicles were identified by the prescence of more than 2 layers of granulosa cells surrounded by the basal lamina and absence of an antrum (Comizzoli, Pukazhenthi & Wildt, 2011).

Follicles were embedded in alginate beads for culture (Xu et al., 2006; Nagashima et al., 2017). First, follicles were washed twice in warmed 0.5% purified alginate (W201502, Sigma Aldrich) dissolved in Mg^2+^- and Ca^2+^-free Dulbecco’s PBS (14,190–144, Gibco). Individual follicles were embedded in alginate beads by dropping 5 µL of media containing the follicle into warmed, sterile-filtered solution of 50 mM CaCl_2_ and140 mM NaCl (Sigma Aldrich). Alginate beads were crosslinked for 2 min before transferring beads to growth media for conditioning.

**Table 3:** Medium composition for follicle culture experiments.

Component	Catalogue number	Media type		
		Transport media	Collection media	Growth media
Base media		MEM with Hank’s Salts [11575-032, Gibco]	MEM with Hank’s Salts [11575-032, Gibco]	MEM with Earle’s Salts [M5650, Sigma-Aldrich]
HEPES buffer	15630-080, Gibco	–	1%	–
Penicillin	P7794, Sigma-Aldrich	100 IU/mL	100 IU/mL	50 IU/mL
Streptomycin	S1277, Sigma-Aldrich	100 µg/mL	100 µg/mL	50 µg/mL
Ascorbic acid	A61-100, Fisher	0.25 mM	–	0.25 mM
L-glutamine	G8540, Sigma Aldrich	–	2 mM	2 mM
ITS+	41400045, Thermofisher	–	–	1%
Bovine serum albumin	A9418, Sigma-Aldrich	–	0.3% w/v	0.3% w/v

Follicle culture was carried out in 96-well plates (353077, Corning). Plates were held in an incubator at 38 °C and 5% CO_2_ throughout experiments except during media changes. Vehicle treatment contained growth medium only (Table 3). FSH-supplemented follicles received 1 µg/mL porcine FSH from aliquoted stock (F2293, Sigma Aldrich). Follicles were exposed to RFRP3 peptide (Cat. No., 048–46, Phoenix Pharmaceuticals Inc., Burlingame, CA) at two doses: 10 µM and 1 µM in sterile PBS. This synthetic version of human RFRP3 is identical in peptide sequence to fRFRP3. Doses were chosen based on treatments used in previous studies (Maddineni et al., 2008; Oishi et al., 2012).

Follicles were individually photographed every 48 h throughout the duration of the experiment (days 0, 2, 4, 6, and 8), and half the volume was changed at the same time (75 µL). All media were conditioned in the incubator prior to addition. Media collected during changes were stored at −80 °C in sterile Eppendorf tubes.

### Follicle integrity and size measurements

Follicle integrity and size were assessed from brightfield photos using ImageJ. Photos were taken on a stereoscope at fixed magnification. Follicles were classified as intact only when their shape was circular and the basal lamina was smooth (representative images shown in Figs. 1A and 1B). Follicles were classed as degraded based on breaks, irregularity, or large gaps in the basal lamina (representative images shown in Figs. 1C–1F). Follicle diameter was measured using two perpendicular line measurements in ImageJ. Follicle size was then calculated as the area of an ellipse using the two diameter measurements. All measurements were collected and recorded by viewers blind to treatment. The average follicle diameter on day 0 was 170.0 ± 40.1 µm (Average ± SD), corresponding to an average area on day 0 of 23.7 × 10^3^ ± 11.2 × 10^3^ µm^2^ (Average ± SD).

### Follicle viability

Follicle viability was assessed in separate cohorts of follicles from those analyzed for effects on follicle integrity and size. Follicle viability was assessed qualitatively using the LIVE/DEAD Viability/Cytotoxicity Kit (L3224, Invitrogen). Concentrations of calcein-AM (Ca-AM) and ethidium homodimer-1(EthD) were optimized prior to use as per the kits instructions. On day 8, only intact follicles embedded in alginate beads were removed from wells and placed in warmed Dulbecco’s PBS containing 6 mg/mL of alginate lyase (A1603, Sigma Aldrich). Alginate was digested for 30 min at 38 °C. Follicles were then washed in warm dPBS and incubated in dPBS contain 4 µM EthD and 1 µM Ca-AM for 30 min. Follicles were cover-slipped and immediately visualized under a fluorescent microscope. Follicles exhibiting any red fluorescence were categorized as dead or dying (inviable), whereas follicles that fluoresced only with green were categorized as alive (viable; See Figs. 1G, 1H for representative images).

### Progesterone extraction and quantification

Progesterone accumulation was assayed using media collected across the culture period (days 2, 4, 6 and 8), which were pooled for each follicle. Steroid hormones were extracted using an ethyl acetate/water wash extraction (*N* = 95). Pooled media (∼300 µL) were mixed with one mL of ethyl acetate (Sigma-Aldrich, 270989) by vigorously vortexing for 5 s. The samples were then mixed on an orbital shaker (700 rpm) for 5 min. Following mixing, layers were separated for 5 min. The organic layer was transferred to one mL MilliQ water, which was mixed and separated identical to the ethyl acetate steps described above. The organic layer was then transferred to a borosilicate glass vial. The procedure was repeated ( one mL ethyl acetate, one mL MilliQ water) and organic layers were combined. Samples were dried under nitrogen stream and stored at −20 °C.

Samples were reconstituted in 150 µL EIA Buffer (Item No. 400060; Cayman Chemical) immediately prior to assay. Samples were assayed using the Cayman Chemical Progesterone ELISA kit (Item No. 582601) according to manufacturer instructions. Samples that fell below the detection limit of the kit (*N* = 4) were assigned the lowest detectable amount on the standard curve (7.81 pg/mL). Samples above the standard curve were diluted (1:10 or 1:41) and reassayed –those samples that still remained above the standard curve after dilution were then assigned the highest detectable value (*N* = 23; 1000 pg/mL) and corrected for dilution factor prior to analysis. The median intra-assay variation was 10.1%, and the inter-assay variation was 22%.

### Statistical approach and analysis and rationale

In total, we embedded 560 follicles isolated from 70 ovaries across six days for our experiments. 242 follicles (30 ovaries) were used for basal lamina degradation and progesterone production experiments. 318 follicles (40 ovaries) were used in the follicle survival experiments. For all isolation days, follicles were evenly assigned to treatments on each day such that all treatments were run for each isolation date. Follicles isolated from each ovary were assigned evenly across the four treatment groups such that each ovary were isolated was represented evenly in each treatment group; note that some ovaries yielded up to 16 follicles used in experiments, whereas others yielded only four. Only isolated follicles with intact basal lamina were embedded for experiments. Experiments and sample sizes are outlined in Fig. 2.

Our experimental design was based on *a priori* predictions, as laid out in the introduction. For this reason, we used planned comparisons for all analyses. We used a vehicle-control and FSH supplementation to determine whether FSH protected follicle viability, as expected based on previous work in this system (Songsasen et al., 2012; Chansaenroj, Songsasen & Chatdarong, 2019). We compared FSH-treatment alone to each concentration of fRFRP3 (1 and 10 µM) in combination with FSH to determine whether fRFRP3 has any effect on proposed outcome measures.

All analyses were completed in R (R Core Team, 2018). We used general linear models (glmer) for most analyses in order to include a random effect of isolation date. We used QQ plots to assess normality and goodness of fit for all models.

In order to determine whether follicle area varied among treatments on day 0, we transformed the dependent variable, follicle size on day 0, using inverse root-square and applied a model with a gaussian distribution. We included treatment as a fixed effect. All follicles were included in the analysis.

Because follicle size could only be determined for follicles with intact basal lamina, follicles that degraded had to be excluded from analyses of the effect of treatment on change in size over the culture period. To determine whether size of intact follicles varied with treatment at the end of the culture period, we transformed the dependent variable, follicle size on day 8, using inverse root-squared and applied a model with a Gaussian distribution. Treatment was included as a fixed effect. To test for effects of treatment on change in size over the period of culture, we again transformed the dependent variable, follicle size, using inverse root-squared and applied a model with a Gaussian distribution. We included a day by treatment interaction and day and treatment independently as fixed effects.

We estimated the effect of treatment on likelihood of loss of follicle integrity using a Cox proportional hazards model, with treatment and starting size as fixed effects.

We estimated the effect of treatment on follicle survival by applying a model with a binomial distribution and logit link. Treatment was included as a fixed effect.

We examined whether size affected progesterone production using Pearson’s product-moment correlation. We log-transformed progesterone concentration for correlation analyses, and we examined change in size using percent of day 0 size $ \left( \left[ 100\times \frac{day8-day0}{day0} \right] +100 \right) $. We estimated the effect of treatment on progesterone production by applying a model with a gamma distribution and log link. Treatment and percent of day 0 size were included as fixed effects. Analyses of the dataset excluding samples that did not fall within the range of the standard curve (*N* = 31) or those with CV > 40% (*N* = 9) yielded qualitatively similar results (reported effects remain significant at *P* < 0.05). For this reason, all samples are included in the results presented here.

**Figure 3:**
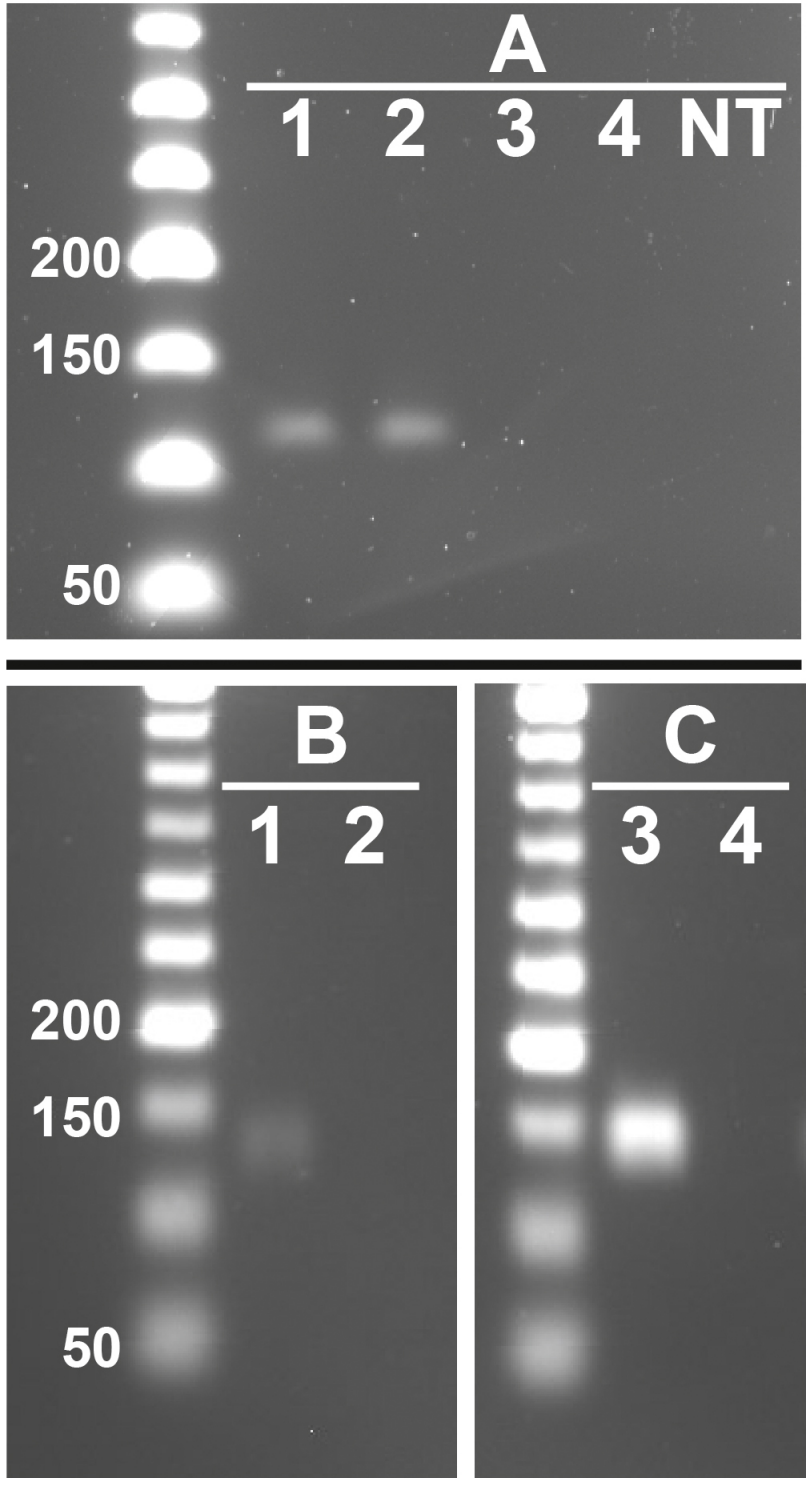
Representative images showing presence of feline RFRP3 (fRFRP3) and receptor transcripts in the domestic cat ovary. fRFRP3 transcripts can be found in RNA isolated from whole ovary (A, lanes 1 and 2), but not in No-RT controls (A, Lane 3 and 4) or no-transcript controls (A, lane NT). The primary RFRP3 receptor (NPFFR1, B) and the secondary receptor (NPFFR2, C) were also found in ovarian tissues (lane 1 & 3). No-RT controls show no amplification (lanes 2 & 4).

**Figure 4:**
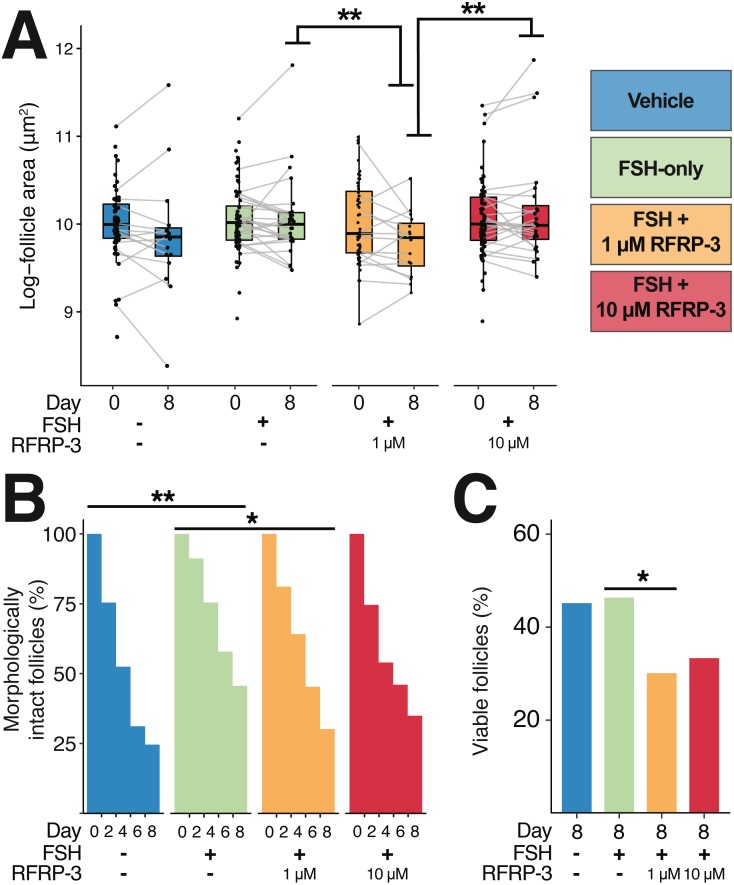
Follicle size, integrity of the basal lamina, and morphology were all affected by 1 µM feline RFRP3 (fRFRP3) treatment. A color-based key to treatments is shown the top right of the figure and along the *x*-axis in (A–C). Isolated follicles were treated with vehicle (blue), 1 µg/mL FSH (green), or 1 µg/mL FSH combined with 1 µM (orange) or 10 µM (red) fRFRP3 throughout the culture period. (A) The size of follicles with intact basal lamina in each experiment for days 0 and 8 of culture. Follicle size (area) was log-transformed for presentation in the boxplot, where each point represents a single follicle’s size on that day. Grey lines connect size measurements on day 0 and day 8 of follicles that maintained basal lamina integrity across the culture period. Lines do not indicate a linear progression of growth or degradation. (B) The percent of ovarian follicles with intact basal lamina every 2 days for the duration of the culture period. (C) The percent of follicles alive on day 8 of treatment based on live/dead fluorescent assay. Asterisks indicate significant differences based on planned comparisons. For (A–C), ^∗∗^*P* < 0.01, ^∗^*P* < 0.05.

## Results

### Presence of fRFRP3 and receptors in ovarian tissues

fRFRP3 was expressed in ovarian tissue of domestic cats (Fig. 3A). Both primary (NPFFR1) and secondary (NPFFR2) fRFRP3 receptors also were expressed in domestic cat ovaries (Figs. 3B, 3C).

### Influence of fRFRP3 on follicle size and integrity

There were no differences in follicle size among treatments at the time of isolation (day 0; Fig. 4A; Table 4). By day 8, intact follicles in the 1 µM fRFRP3 treatment were significantly smaller than intact follicles in the FSH-only and the 10 µM treatment (Fig. 4A; Table 4). However, there was no effect of culture period on change in follicle size over time (*t*_3096_ = 1.05, *P* = 0.29) or interaction between time and treatment (*P* > 0.61 for all comparisons) for follicles that maintained integrity. The difference in size on day 8 appears to be a selective effect of 1 µM RFRP3 on larger follicles: when we compared follicle size on day 0 among only those that retained integrity across the culture period, follicles that persisted in the 1 µM fRFRP3 treatment were smaller compared to those that persisted in the FSH-only or the 10 µM group (Table 4).

**Table 4:** Summary of statistical test statistics and *p*-values from analyses. Significant *p*-values are bolded and italicized.

	Vehicle vs. FSH			FSH vs. 1 µM			FSH vs. 10 µM			1 µM vs. 10 µ*M*		
	Test statistic	DF	*p*-value	Test statistic	DF	*p*-value	Test statistic	DF	*p*-value	Test statistic	DF	*p*-value
Follicle size, day 0	*t* = 0.24	247.13	0.81	*t* = 0.11	247.2	0.91	*t* = 0.74	246.9	0.46	*t* = 0.58	247.3	0.56
Follicle size, day 8	*t* = 1.80	74.9	0.07	*t* = − 2.19	***75.6***	***0.03***	*t* = − 0.32	75.15	0.75	*t* = − 2.43	***75.6***	***0.02***
Follicle size, day 0, only follicles that maintain integrity for 8 days	*t* = 1.57	75.2	0.12	*t* = − 2.07	***76.42***	***0.041***	*t* = − 0.06	75.5	0.95	*t* = − 2.09	***76.41***	***0.040***
Degradation likelihood	***z = 2.62***	–	***0.009***	***z = 2.04***	–	***0.04***	*z* = 1.33	–	0.18	*z* = 0.080	–	0.42
Follicle Survival	*z* = 0.14	–	0.88	*z* = − 2.20	–	***0.03***	*z* = 1.69	–	0.09	*z* = − 0.46	–	0.65
Progesterone accumulation	*t* = 0.91	91	0.36	***t = 3.07***	***91***	***0.002***	*t* = − 0.76	91	0.44	*t* = − 3.83	***91***	***0.0001***

The likelihood of degradation was lower in FSH-treated follicles relative to vehicle-controls (Fig. 4B; Table 4). The 1 µM fRFRP3 treatment increased the likelihood of basal lamina degradation relative to FSH-treated follicles (Fig. 4B; Table 4). The likelihood of basal lamination degradation in 10 µM treatment did not significantly differ from FSH-only or 1 µM fRFRP3 treated follicles (Fig. 4B; Table 4).

### Influence of fRFRP3 on follicle viability

There was no effect of FSH supplementation on cell viability relative to vehicle-controls (*z* = 0.141, *P* = 0.88). However, the 1 µM fRFRP3 treatment decreased follicle viability (Fig. 4C; Table 4). There was no difference in viability between 10 µM fRFRP3 treatment and FSH-only or 1 µM fRFRP3 treated follicles (Fig. 4C; Table 4).

**Figure 5:**
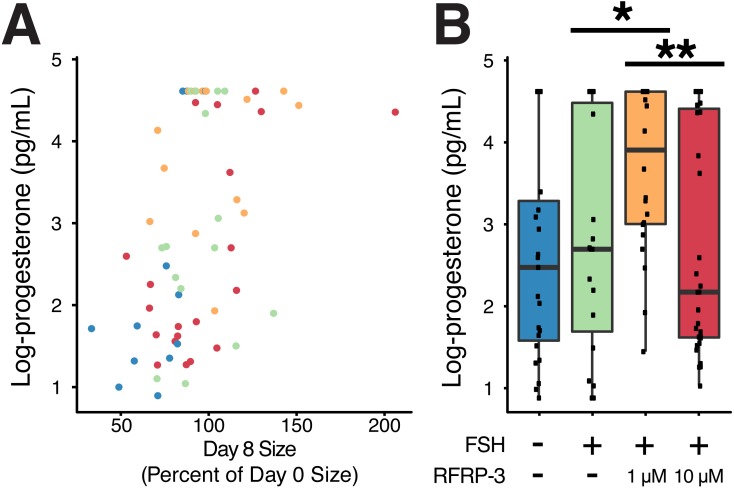
Cumulative progesterone production by individual follicles across the culture period was related to change in size and affected by treatment. Isolated follicles were treated with vehicle (blue), 1 µg/mL FSH (green), or 1 µg/mL FSH combined with 1 µM (orange) or 10 µM (red) fRFRP3 throughout the culture period. (A) Size of follicles on day 8 (as a percent of their size on day 0) was positively correlated with progesterone production over the culture period (*P* < 0.0001). Each point represents a single, unique follicle (B) Progesterone concentration varied among treatment groups. Progesterone was log-transformed for presentation in the boxplot, where each point represents total progesterone from an individual follicle. Asterisks indicate significant differences based on planned comparisons. ^∗∗^*P* < 0.01, ^∗^*P* < 0.05.

### Influence of fRFRP3 on progesterone production

Size of the follicle at the start of culture had no relationship to progesterone production over the culture period (*t*_91_ =  − 0.18, *P* = 0.85), however there was a positive relationship between change in size of the follicle over culture and progesterone production (*t*_61_ = 3.97, *P* < 0.0002, *R*^2^ = 0.42; Fig. 5A).

FSH supplementation did not modify progesterone production relative to vehicle-controls (Table 4). However, addition of 1 µM fRFRP3 resulted in increased production of progesterone relative to FSH-only and 10 µM fRFRP3 treatments (Fig. 5B; Table 4). There was no difference in progesterone production between 10 µM fRFRP3 and FSH-only treated follicles (Fig. 5B; Table 4).

## Discussion

Our study is the first to examine effects of RFRP3 on the isolated follicle as a functional unit, and it is the first study of RFRP3 expression and function in any carnivore species. We demonstrate that the domestic cat ovary produces fRFRP3 and its receptors. Furthermore, we show that fRFRP3 can influence the viability, integrity, and steroid production of isolated domestic cat follicles *in vitro*.

We were able to identify transcripts for the fRFRP3 peptide in the domestic cat ovary; our data are thus consistent with the hypothesis that there is conserved expression of the fRFRP3 peptides in the vertebrate ovary. We also found expression of both receptors for fRFRP3, NPFFR1 and NPFFR2, in the ovary of the domestic cat. To our knowledge, no other study has yet examined presence/absence or distribution of the NPFFR2 receptor in the ovaries. Although RFRP3 has a greater binding affinity for NPFFR1 (Gouardères et al., 2007), others have suggested that modulating the relative abundance of these two receptors in the same tissue could allow RFRP3 to regulate distinct functions. Both receptors are also found in the testes of Syrian hamsters (Zhao et al., 2010), so we expect that both receptors are likely to be present in the ovaries of other species. In Syrian hamster testes, NPFFR1 was localized to spermatids at all stages of spermatogenesis, whereas NPFFR2 expression was limited to late elongated spermatids (Zhao et al., 2010), leading Zhao et al. (2010) to suggest that RFRP3 may act on these receptors to regulate related but distinct processes in spermatogenesis. To determine whether similar hypotheses can be extended to direct action on oocyte maturation, future studies will need to combine localization and targeted functional (receptor-specific antagonists/agonists, Kim et al., 2015) studies.

In order to assess functional effects of fRFRP3 on follicle viability, we quantified morphological integrity, follicle size, and viability over the course of treatment. We found no effect of FSH or fRFRP3 exposure on follicle change in size during the study period. However, because we focused on early-stage follicles, longer periods of culture may be needed to generate measurable changes in follicle size. We did find that exposure to 1 µM, but not 10 µM, of fRFRP3 increased the proportion of morphologically degraded follicles over time (Fig. 4B) and increased the proportion of follicles exhibiting cellular death (Fig. 4C). Moreover, the decrease in follicle size in the 1 µM fRFRP3 treatment group suggests that there is selective loss of larger early-stage follicles or decrease in size in response to 1 µM fRFRP3. Together, these findings suggest that fRFRP3 could inhibit maturation and viability of early-stage follicles. Given that the receptor for RFRP3 is often found to be more abundant in early-stage follicles (Maddineni et al., 2008; Qi et al., 2013), whereas the RFRP3 peptide is more abundant in tertiary or pre-ovulatory follicles (Singh et al., 2008; Singh et al., 2011; Oishi et al., 2012; Squicciarini et al., 2018) (see Table 1 for some exceptions), we speculate that RFRP3 production by tertiary follicles suppresses the maturation of early-stage by promoting follicle degradation through paracrine signaling.

We also found that the 1 µM fRFRP3 *increased* progesterone production by domestic cat follicles, whereas RFRP3 has been shown to *decrease* steroid production by the ovaries in all other studies to-date (see Table 1). There are several potential explanations for this discrepancy which highlight the need for more comparative, functional experimentation. First, our results may differ from others published to date because we are the first to examine action of RFRP3 on the follicle as a functional unit, whereas previous work has focused on isolated granulosa cells or whole ovaries (Table 1). Apparent effects of RFRP3 on the whole follicle may differ from effects measured at the whole ovary or on isolated granulosa cells because of the extensive role endocrine and paracrine signaling play in regulating follicle development, degradation, and ovulation. In our study, the increase in progesterone production could be a function of decreased utilization of pregnenolone by granulosa cells for synthesis of estradiol and other sex steroids. Quantifying production of multiple steroids is needed to resolve this question. Pairing such data with gene expression or enzyme activity would be useful for determining the mechanism underlying the increase in progesterone production we show. Our results may also be specific to preantral follicles, whereas previous studies have focused on cells isolated from hierarchical (hierarchical and pre-ovulatory) follicles. Experiments that explore a broader range of follicle classes are therefore needed to determine whether effects of RFRP3 on steroid production is stage-specific.

Second, the elevated progesterone production by domestic cat follicles treated with 1 µM fRFRP-3 could be a result of the environmental conditions that cats experienced prior to spaying; the environmental context may determine the capacity for and/or direction of the ovarian follicles’ responses to fRFRP3. RFRP3 action is context-dependent in other seasonal breeders, including Siberian hamsters (Ubuka et al., 2012) and European Starlings (McGuire, Kangas & Bentley, 2011). In Siberian hamsters, RFRP3 inhibits gonadotropin release under long-day photoperiods (breeding conditions), but it promotes gonadotropin release under short-day photoperiods (non-breeding, Ubuka et al., 2012). In European Starlings, the inhibitory action of RFRP3 on testicular testosterone secretion occurs only prior to the onset of breeding –testicular testosterone production of breeding birds is insensitive to RFRP3 (McGuire, Kangas & Bentley, 2011). Domestic cats breed seasonally (Spindler & Wildt, 1999; Blottner & Jewgenow, 2007; Faya et al., 2011), and feral females do not become pregnant between November and early February, even in mild climates (K Wilsterman, 2014–2018, per. obs.). Tissues used in this study were collected from feral cats during the spring –follicles may respond differently to fRFRP3 if experiments were carried out at other times of the year. Contrasting results from different periods within known breeding cycles is necessary to determine whether ovarian RFRP3 exerts context-dependent action.

Third and finally, it is also possible that the apparently unique stimulation of steroid production reflects species-specific or carnivore-specific function of the fRFRP3 system. Similar species-specific action of RFRP3 has been demonstrated in the hypothalamus and ovary in other species. In the Syrian hamster, RFRP3 stimulates hypothalamic GnRH instead of inhibiting hypothalamic GnRH, as is the case in most vertebrates (Ancel et al., 2012), and in the grouper ovary, RFRP3 promotes transcription of steroidogenic enzymes and luteinizing hormone receptor (LHR; Wang et al., 2017). Although RFRP3 does exert remarkably conserved action in the reproductive system of many vertebrates, it is also clear that there is species-specific variation in. Because felids display unique diversity in reproductive function (Pelican et al., 2006), we also might expect to see greater diversity or idiosyncrasy of peptide function. Understanding the evolutionary drivers (e.g., natural selection, genetic drift) of variation in reproductive system function among felids is largely an open but exciting question.

Taken together, our results are broadly consistent with the functional and observational studies conducted to-date, and they support the potential for RFRP3 to serve as a useful target for development of tools related to feral and invasive species management and endangered species survival plans. Ultimately, effects of RFRP3 on follicle viability will be more important for species management than will the effects on steroid production alone, and our results support that RFRP3 may decrease follicle viability. Realizing the utility of RFRP3 for applications in population management will depend on developing a better understanding the molecular pathways underlying RFRP3-mediated degradation of follicle integrity and any potential dose-dependency of these effects. We used concentrations of fRFRP3 at the upper range of those found to be effective in other studies because alginate embedding can decrease the rate of protein diffusion to the follicles in culture (Heise et al., 2005; West, Shea & Woodruff, 2007), however our findings suggest that the higher concentrations used here might not be biologically or pharmacologically-relevant. More conservative (lower) concentrations are likely to be more effective for future functional studies in felines.

More broadly, our finding that RFRP3 decreases follicle integrity and cell viability is consistent with the inhibitory effect of RFRP3 on reproductive function of the ovary in other mammals and in birds. These results thus add credence to a conserved effect of RFRP3 on vertebrate ovary function. The broad concordance in function should encourage comparative research into the function of RFRP3 across the reproductive axis in vertebrates. Because the conservation of expression does not guarantee conservation of function, functional studies are more likely to rapidly advance our understanding of RFRP3 functional evolution and conservation.

## Conclusions

Though there is interest in the application of RFRP3 to human reproductive diseases (Squicciarini et al., 2018), animal control (Rhodes, 2017; McCormick & Romero, 2017), and endangered species survival programs (Comizzoli & Holt, 2019), its potential in these arenas depends on functional studies that will determine species-specific dose-dependency, gonadotropin-sensitivity, and efficacy of action at the level of the whole ovary. In addition to focusing on RFRP3, the regulation and function of the two receptors for the RFRP3 peptide (NPFFR1 and NPFF2) needs to be elucidated to complete our understanding of this system. RFRP3 provides a unique opportunity to study the basic evolution of hormone systems in central and peripheral reproductive systems while simultaneously advancing development of applied technologies.

##  Supplemental Information

10.7717/peerj.7540/supp-1 Supplemental Information 1 METADATA Metadata for datasets associated with this publication.

10.7717/peerj.7540/supp-2 Supplemental Information 2 Follicle Integrity Data

10.7717/peerj.7540/supp-3 Supplemental Information 3 Follicle Size and Degradation Dataset

10.7717/peerj.7540/supp-4 Supplemental Information 4 Progesterone Data

10.7717/peerj.7540/supp-5 Supplemental Information 5 Follicle Viability Data

## Additional Information and Declarations

### Data Availability

The following information was supplied regarding data availability: Raw data used in analyses are available in the Supplemental Files.

## References

[ref-1] Ancel C, Bentsen AH, Sébert M-E, Tena-Sempere M, Mikkelsen JD, Simonneaux V (2012). Stimulatory effect of RFRP-3 on the gonadotrophic axis in the male syrian hamster: the exception proves the rule. Endocrinology.

[ref-2] Anderson GM, Relf H-L, Rizwan MZ, Evans JJ (2009). Central and peripheral effects of RFamide-related peptide-3 on luteinizing hormone and prolactin secretion in rats. Endocrinology.

[ref-3] Bentley GE, Tsutsui K, Kriegsfeld LJ (2010). Recent studies of gonadotropin-inhibitory hormone (GnIH) in the mammalian hypothalamus, pituitary and gonads. Brain Research.

[ref-4] Bentley GE, Ubuka T, McGuire NL, Chowdhury VS, Morita Y, Yano T, Hasunuma I, Binns M, Wingfield JC, Tsutsui K (2008). Gonadotropin-inhibitory hormone and its receptor in the avian reproductive system. General and Comparative Endocrinology.

[ref-5] Bentley GE, Wilsterman K, Ernst DK, Lynn SE, Dickens MJ, Calisi RM, Kriegsfeld LJ, Kaufer D, Geraghty AC, viviD D, McGuire NL, Lopes PC, Tsutsui K (2017). Neural versus gonadal GnIH: are they independent systems? A mini-review. Integrative and Comparative Biology.

[ref-6] Biran J, Golan M, Mizrahi N, Ogawa S, Parhar IS, Levavi-Sivan B (2014). LPXRFa, the piscine ortholog of GnIH, and LPXRF receptor positively regulate gonadotropin secretion in Tilapia (Oreochromis niloticus). Endocrinology.

[ref-7] Blottner S, Jewgenow K (2007). Moderate seasonality in testis function of domestic cat. Reproduction in Domestic Animals.

[ref-8] Chansaenroj A, Songsasen N, Chatdarong K (2019). Equine chorionic gonadotropin induces in vitro follicular growth from the multi-layered secondary developmental stage in cats. Theriogenology.

[ref-9] Clarke IJ, Sari IP, Qi Y, Smith JT, Parkington HC, Ubuka T, Iqbal J, Li Q, Tilbrook A, Morgan K, Pawson AJ, Tsutsui K, Millar RP, Bentley GE (2008). Potent action of RFamide-related peptide-3 on pituitary gonadotropes indicative of a hypophysiotropic role in the negative regulation of gonadotropin secretion. Endocrinology.

[ref-10] Comizzoli P, Holt WV (2019). Breakthroughs and new horizons in reproductive biology of rare and endangered animal species. Biology of Reproduction.

[ref-11] Comizzoli P, Pukazhenthi BS, Wildt DE (2011). The competence of germinal vesicle oocytes is unrelated to nuclear chromatin configuration and strictly depends on cytoplasmic quantity and quality in the cat model. Human Reproduction.

[ref-12] Corchuelo S, Martinez ERM, Butzge AJ, Doretto LB, Ricci JMB, Valentin FN, Nakaghi LSO, Somoza GM, Nóbrega RH (2017). Characterization of Gnrh/Gnih elements in the olfacto-retinal system and ovary during zebrafish ovarian maturation. Molecular and Cellular Endocrinology.

[ref-13] Ducret E, Anderson GM, Herbison AE (2009). RFamide-related peptide-3, a mammalian gonadotropin-inhibitory hormone ortholog, regulates gonadotropin-releasing hormone neuron firing in the mouse. Endocrinology.

[ref-14] Ernst DK, Lynn SE, Bentley GE (2016). Differential response of GnIH in the brain and gonads following acute stress in a songbird. General and Comparative Endocrinology.

[ref-15] Fang MX, Huang YS, Ye J, Zhang W, Li Y, Nie QH (2014). Identification and characterization of RFRP gene in pigs and its association with reproductive traits. Genetics and Molecular Research.

[ref-16] Faya M, Carranza A, Priotto M, Abeya M, Diaz JD, Gobello C (2011). Domestic queens under natural temperate photoperiod do not manifest seasonal anestrus. Animal Reproduction Science.

[ref-17] Gouardères C, Mazarguil H, Mollereau C, Chartrel N, Leprince J, Vaudry H, Zajac J-M (2007). Functional differences between NPFF1 and NPFF2 receptor coupling: High intrinsic activities of RFamide-related peptides on stimulation of [35S]GTP*γ*S binding. Neuropharmacology.

[ref-18] Heise M, Koepsel R, Russell AJ, McGee EA (2005). Calcium alginate microencapsulation of ovarian follicles impacts FSH delivery and follicle morphology. Reproductive Biology and Endocrinology.

[ref-19] Herrick JR (2019). Assisted reproductive technologies for endangered species conservation: developing sophisticated protocols with limited access to animals with unique reproductive mechanisms. Biology of Reproduction.

[ref-20] Iwakoshi-Ukena E, Ukena K, Takuwa-Kuroda K, Kanda A, Tsutsui K, Minakata H (2004). Expression and distribution of octopus gonadotropin-releasing hormone in the central nervous system and peripheral organs of the octopus (Octopus vulgaris) by in situ hybridization and immunohistochemistry. The Journal of Comparative Neurology.

[ref-21] Kadokawa H, Shibata M, Tanaka Y, Kojima T, Matsumoto K, Oshima K, Yamamoto N (2009). Bovine C-terminal octapeptide of RFamide-related peptide-3 suppresses luteinizing hormone (LH) secretion from the pituitary as well as pulsatile LH secretion in bovines. Domestic Animal Endocrinology.

[ref-22] Kim JS, Brownjohn PW, Dyer BS, Beltramo M, Walker CS, Hay DL, Painter GF, Tyndall JDA, Anderson GM (2015). Anxiogenic and stressor effects of the hypothalamic neuropeptide RFRP-3 are overcome by the NPFFR antagonist GJ14. Endocrinology.

[ref-23] Kriegsfeld LJ, Jennings KJ, Bentley GE, Tsutsui K (2018). Gonadotrophin-inhibitory hormone and its mammalian orthologue RFamide-related peptide-3: discovery and functional implications for reproduction and stress. Journal of Neuroendocrinology.

[ref-24] Kriegsfeld LJ, Mei DF, Bentley GE, Ubuka T, Mason AO, Inoue K, Ukena K, Tsutsui K, Silver R (2006). Identification and characterization of a gonadotropin-inhibitory system in the brains of mammals. Proceedings of the National Academy of Sciences of the United States of America.

[ref-25] Li H, Song H, Huang M, Nie H, Wang Z, Wang F (2014). Impact of food restriction on ovarian development, RFamide-related peptide-3 and the hypothalamic-pituitary-ovarian axis in pre-pubertal ewes. Reproduction in Domestic Animals = Zuchthygiene.

[ref-26] Li X, Su J, Fang R, Zheng L, Lei R, Wang X, Lei Z, Jin M, Jiao Y, Hou Y, Guo T, Ma Z (2013). The effects of RFRP-3, the mammalian ortholog of GnIH, on the female pig reproductive axis in vitro. Molecular and Cellular Endocrinology.

[ref-27] Li X, Su J, Lei Z, Zhao Y, Jin M, Fang R, Zheng L, Jiao Y (2012). Gonadotropin-inhibitory hormone (GnIH) and its receptor in the female pig: cDNA cloning, expression in tissues and expression pattern in the reproductive axis during the estrous cycle. Peptides.

[ref-28] Maddineni SR, Ocón-Grove OM, Krzysik-Walker SM, Hendricks GL, Ramachandran R (2008). Gonadotropin-inhibitory hormone (GnIH) receptor gene is expressed in the chicken ovary: potential role of GnIH in follicular maturation. Reproduction.

[ref-29] McCormick SD, Romero LM (2017). Conservation endocrinology. BioScience.

[ref-30] McGuire NL, Bentley GE (2010). Neuropeptides in the gonads: from evolution to pharmacology. Frontiers in Pharmacology.

[ref-31] McGuire NL, Kangas K, Bentley GE (2011). Effects of melatonin on peripheral reproductive function: regulation of testicular GnIH and testosterone. Endocrinology.

[ref-32] McGuire NL, Koh A, Bentley GE (2013). The direct response of the gonads to cues of stress in a temperate songbird species is season-dependent. PeerJ.

[ref-33] Medina FM, Bonnaud E, Vidal E, Tershy BR, Zavaleta ES, Donlan CJ, Keitt BS, Corre ML, Horwath SV, Nogales M (2011). A global review of the impacts of invasive cats on island endangered vertebrates. Global Change Biology.

[ref-34] Murakami M, Matsuzaki T, Iwasa T, Yasui T, Irahara M, Osugi T, Tsutsui K (2008). Hypophysiotropic role of RFamide-related peptide-3 in the inhibition of LH secretion in female rats. The Journal of Endocrinology.

[ref-35] Nagashima J, Wildt DE, Travis AJ, Songsasen N (2017). Follicular size and stage and gonadotropin concentration affect alginate-encapsulated in vitro growth and survival of pre- and early antral dog follicles. Reproduction, Fertility and Development.

[ref-36] Oishi H, Klausen C, Bentley GE, Osugi T, Tsutsui K, Gilks CB, Yano T, Leung PCK (2012). The human gonadotropin-inhibitory hormone ortholog RFamide-related peptide-3 suppresses gonadotropin-induced progesterone production in human granulosa cells. Endocrinology.

[ref-37] Osugi T, Daukss D, Gazda K, Ubuka T, Kosugi T, Nozaki M, Sower SA, Tsutsui K (2012). Evolutionary origin of the structure and function of gonadotropin-inhibitory hormone: insights from Lampreys. Endocrinology.

[ref-38] Osugi T, Ukena K, Bentley GE, O’Brien S, Moore IT, Wingfield JC, Tsutsui K (2004). Gonadotropin-inhibitory hormone in Gambel’s white-crowned sparrow (Zonotrichia leucophrys gambelii): cDNA identification, transcript localization and functional effects in laboratory and field experiments. The Journal of Endocrinology.

[ref-39] Paullada-Salmerón JA, Cowan M, Aliaga-Guerrero M, Gómez A, Zanuy S, Mañanos E, Muñoz Cueto JA (2016). LPXRFa peptide system in the European sea bass: a molecular and immunohistochemical approach. Journal of Comparative Neurology.

[ref-40] Pelican KM, Wildt DE, Pukazhenthi B, Howard J (2006). Ovarian control for assisted reproduction in the domestic cat and wild felids. Theriogenology.

[ref-41] Qi X, Zhou W, Lu D, Wang Q, Zhang H, Li S, Liu X, Zhang Y, Lin H (2013). Sexual dimorphism of steroidogenesis regulated by GnIH in the Goldfish, Carassius auratus. Biology of Reproduction.

[ref-42] R Core Team (2018). https://www.r-project.org.

[ref-43] Rhodes L (2017). New approaches to non-surgical sterilization for dogs and cats: opportunities and challenges. Reproduction in Domestic Animals.

[ref-44] Shahjahan M, Ikegami T, Osugi T, Ukena K, Doi H, Hattori A, Tsutsui K, Ando H (2011). Synchronised expressions of LPXRFamide peptide and its receptor genes: seasonal, diurnal and circadian changes during spawning period in grass puffer. Journal of Neuroendocrinology.

[ref-45] Singh P, Krishna A, Sridaran R, Tsutsui K (2008). Changes in GnRH I. bradykinin and their receptors and GnIH in the ovary of Calotes versicolor during reproductive cycle. General and Comparative Endocrinology.

[ref-46] Singh P, Krishna A, Sridaran R, Tsutsui K (2011). Immunohistochemical localization of GnRH and RFamide-related peptide-3 in the ovaries of mice during the estrous cycle. Journal of Molecular Histology.

[ref-47] Singh P, Krishna A, Tsutsui K (2011). Effects of gonadotropin-inhibitory hormone on folliculogenesis and steroidogenesis of cyclic mice. Fertility and Sterility.

[ref-48] Son YL, Ubuka T, Soga T, Yamamoto K, Bentley GE, Tsutsui K (2016). Inhibitory action of gonadotropin-inhibitory hormone on the signaling pathways induced by kisspeptin and vasoactive intestinal polypeptide in GnRH neuronal cell line, GT1-7. The FASEB Journal.

[ref-49] Songsasen N, Comizzoli P, Nagashima J, Fujihara M, Wildt DE (2012). The domestic dog and cat as models for understanding the regulation of ovarian follicle development in vitro. Reproduction in Domestic Animals.

[ref-50] Spindler RE, Wildt DE (1999). Circannual variations in intraovarian oocyte but not epididymal sperm quality in the domestic cat. Biology of Reproduction.

[ref-51] Squicciarini V, Riquelme R, Wilsterman K, Bentley GE, Lara HE (2018). Role of RFRP-3 in the development of cold stress-induced polycystic ovary phenotype in rats. Journal of Endocrinology.

[ref-52] Thongkittidilok C, Singh RP, Comizzoli P, Wildt D, Songsasen N (2018). Insulin promotes preantral follicle growth and antrum formation through temporal expression of genes regulating steroidogenesis and water transport in the cat. Reproduction, Fertility and Development.

[ref-53] Tsutsui K, Bentley GE, Ciccone N, Dawson A, Sharp PJ (2015). Structure, action and functional significance of GnIH. Functional Avian Endocrinology.

[ref-54] Tsutsui K, Saigoh E, Ukena K, Teranishi H, Fujisawa Y, Kikuchi M, Ishii S, Sharp PJ (2000). A novel avian hypothalamic peptide inhibiting gonadotropin release. Biochemical and Biophysical Research Communications.

[ref-55] Tsutsui K, Ubuka T, Bentley GE, Kriegsfeld LJ (2012). Gonadotropin-inhibitory hormone (GnIH): Discovery, progress and prospect. General and Comparative Endocrinology.

[ref-56] Tsutsui K, Ubuka T, Yin H, Osugi T, Ukena K, Bentley GE, Ciccone N, Inoue K, Chowdhury VS, Sharp PJ, Wingfield JC (2006). Mode of action and functional significance of avian gonadotropin-inhibitory hormone (GnIH): a review. Journal of Experimental Zoology. Part A. Comparative Experimental Biology.

[ref-57] Ubuka T, Inoue K, Fukuda Y, Mizuno T, Ukena K, Kriegsfeld LJ, Tsutsui K (2012). Identification, expression, and physiological functions of siberian hamster gonadotropin-inhibitory hormone. Endocrinology.

[ref-58] Wang B, Liu Q, Liu X, Xu Y, Shi B (2018). Molecular characterization and expression profiles of LPXRFa at the brain-pituitary-gonad axis of half-smooth tongue sole (Cynoglossus semilaevis) during ovarian maturation. Comparative Biochemistry and Physiology. Part B. Biochemistry & Molecular Biology.

[ref-59] Wang Q, Qi X, Tang H, Guo Y, Li S, Li G, Yang X, Zhang H, Liu X, Lin H (2017). Molecular identification of StAR and 3βHSD1 and characterization in response to GnIH stimulation in protogynous hermaphroditic grouper (Epinephelus coioides). Comparative Biochemistry and Physiology. Part B. Biochemistry & Molecular Biology.

[ref-60] Wang X, Li X, Hu C (2018). RFRP-3, the mammalian ortholog of GnIH, induces cell cycle arrest at G2/M in porcine ovarian granulosa cells. Peptides.

[ref-61] West ER, Shea LD, Woodruff TK (2007). Engineering the follicle microenvironment. Seminars in Reproductive Medicine.

[ref-62] Xu M, West E, Shea LD, Woodruff TK (2006). Identification of a stage-specific permissive in vitro culture environment for follicle growth and oocyte development. Biology of Reproduction.

[ref-63] Zhang Y, Li S, Liu Y, Lu D, Chen H, Huang X, Liu X, Meng Z, Lin H, Cheng CHK (2010). Structural diversity of the GnIH/GnIH receptor system in teleost: its involvement in early development and the negative control of LH release. Peptides.

[ref-64] Zhao S, Zhu E, Yang C, Bentley GE, Tsutsui K, Kriegsfeld LJ (2010). RFamide-related peptide and messenger ribonucleic acid expression in mammalian testis: association with the spermatogenic cycle. Endocrinology.

