# Supplementary material for: RFRP3 influences basal lamina degradation, cellular death, and progesterone secretion in cultured preantral ovarian follicles from the domestic cat
Source: PeerJ. 2019 Aug 23;7:e7540. doi: 10.7717/peerj.7540 (PMC6709664; doi:10.7717/peerj.7540)
Supplement: Supplemental Information 1 — Metadata for datasets associated with this publication. [file peerj-07-7540-s001.pdf]

Metadata for supplemental datasets

Dataset: BasalLaminaDegradationDataset
Each row is a unique follicle used in experiments.

Column descriptions

Plate: ID of 96-well plate in which experiments were run (A-H)

Well: Well within 96-well plate in which follicle was cultures (B2-G11)

TRT: Treatment group (CON – vehicle control; FSH – 1 ug/mL FSH; LOW – 1 ug/mL FSH + 1 um fRFRP-3; HIGH - 1 ug/mL FSH + 10 um fRFRP-3

IsoDOY: Day of the year on which follicles were isolated

CLASS: Class categorization of follicle type (all follicles used are preantral)

0.Q-8.Q: Quality score for follicle on days 0 through 8. (1, intact; 0, degraded)

DAY: Day on which follicle was observed as degraded (2, 4, 6, or 8)

EVENT: Occurrence of degradation (1, follicle degraded during culture; 0, follicle remained intact across culture period)

Censor: Inverse of Event

L_day: Last day follicle was observed to be intact

SIZE: Area of the follicle on day 0 (um^2)

Dataset: FollicleSizeDataset
Each row is a unique observation of a follicle on day 0 or 8.

Column descriptions

Plate: ID of 96-well plate in which experiments were run (A-H)

Well: Well within 96-well plate in which follicle was cultures (B2-G11)

TRT: Treatment group (CON – vehicle control; FSH – 1 ug/mL FSH; LOW – 1 ug/mL FSH + 1 um fRFRP-3; HIGH - 1 ug/mL FSH + 10 um fRFRP-3

IsoDOY: Day of the year on which follicles were isolated

DAY: Day of observation (0, 8)

SIZE: Area of the follicle (um^2)

lgS: log-transformed area of the follicle (um^2)

Dataset: ProgesteroneData
Each row is progesterone data from a unique follicle used in experiments.

Column descriptions

ID: Numeric ID assigned during extractions

CPLATE: ID of 96-well plate in which experiments were run (A-H)

Well: Well within 96-well plate in which follicle was cultures (B2-G11)

TRT: Treatment group (CON – vehicle control; FSH – 1 ug/mL FSH; LOW – 1 ug/mL FSH + 1 um fRFRP-3; HIGH - 1 ug/mL FSH + 10 um fRFRP-3

Round: Extraction round

Date: Extraction date

Iso_NOTES: Qualitative notes on the extraction process, including any potential loss of sample

EIA_notes: Notes on samples falling above or below limit of detection during ELISA

CV: coefficient of variation among duplicate wells in EIA

CorrValue: Concentration of progesterone measured in EIA

precise: Yes/no whether CorrValue is exact or from limits of standard curve

day0size: Area of the follicle on day 0 (um^2)

day8size: Area of the follicle on day 8(um^2)

absdsize: Percent change in size over culture period ($\frac{100\times[day\ 8 - day\ 0]}{day\ 0} + 100$)

Dataset: LiveDeadData

Each row is counts of follicles in a given outcome and treatment group.

<u>Column descriptions</u>

Iteration: Round of experiment

TRT: Treatment group (CON – vehicle control; FSH – 1 ug/mL FSH; 1um – 1 ug/mL FSH + 1 um fRFRP-3; 10um - 1 ug/mL FSH + 10 um fRFRP-3

outcome: Staining label (live, mix, dead) – **<u>note:</u>** for analyses, mix and dead were combined

N: Count of follicles categorized in this outcome based on fluorescent markers
